# Effectiveness of Proadrenomedullin Enhanced CURB65 Score Algorithm in Patients with Community-Acquired Pneumonia in “Real Life”, an Observational Quality Control Survey

**DOI:** 10.3390/jcm3010267

**Published:** 2014-03-14

**Authors:** Daniel Widmer, Daniel Drozdov, Kristina Rüegger, Alexander Litke, Birsen Arici, Katharina Regez, Merih Guglielmetti, Ursula Schild, Antoinette Conca, Petra Schäfer, Rita Bossart Kouegbe, Barbara Reutlinger, Claudine Blum, Philipp Schuetz, Sarosh Irani, Andreas Huber, Ulrich Bürgi, Beat Müller, Werner C. Albrich

**Affiliations:** 1 Medical University Department, University of Basel, Kantonsspital Aarau, Tellstrasse, Aarau 5001, Switzerland; E-Mails: dany.widmer@gmx.ch (D.W.); daniel.drozdov@ksa.ch (D.D.); kristina.rueegger@unibas.ch (K.R.); alexander.litke@ksa.ch (A.L.); birsen.arici@unibas.ch (B.A.); katharina.regez@ksa.ch (K.R.); merih.guglielmetti@ksa.ch (M.G.); ursula.schild@ksa.ch (U.S.); rita.bossartkouegbe@ksa.ch (R.B.K.); claudine.blum@ksa.ch (C.B.); philipp.schuetz@ksa.ch (P.S.); beat.mueller@ksa.ch (B.M.); 2 Department of Clinical Nursing Science, Kantonsspital Aarau, Tellstrasse, Aarau 5001, Switzerland; E-Mails: antoinette.conca@ksa.ch (A.C.); petra.schaefer-keller@alumnibasel.ch (P.S.); barbara.reutlinger@ksa.ch (B.R.); 3 Division of Pulmonary Medicine, Kantonsspital Aarau, Tellstrasse, Aarau 5001, Switzerland; E-Mail: sarosh.irani@ksa.ch; 4 Department of Laboratory Medicine, Kantonsspital Aarau, Tellstrasse, Aarau 5001, Switzerland; E-Mail: andreas.huber@ksa.ch; 5 Department of Emergency Medicine, Kantonsspital Aarau, Tellstrasse, Aarau 5001, Switzerland; E-Mail: ulrich.buergi@ksa.ch; 6 Division of Infectious Diseases and Hospital Hygiene, Cantonal Hospital St. Gallen, Rorschacherstrasse 95, St. Gallen CH-9007, Switzerland

**Keywords:** biomarkers, proadrenomedullin, clinical scores, CURB65, pneumonia

## Abstract

**Background:** An intervention trial found a trend for shorter length of stay (LOS) in patients with community-acquired pneumonia (CAP) when the CURB65 score was combined with the prognostic biomarker proadrenomedullin (ProADM) (CURB65-A). However, the efficacy and safety of CURB65-A in real life situations remains unclear. **Methods:** From September, 2011, until April, 2012, we performed a post-study prospective observational quality control survey at the cantonal Hospital of Aarau, Switzerland of consecutive adults with CAP. The primary endpoint was length of stay (LOS) during the index hospitalization and within 30 days. We compared the results with two well-defined historic cohorts of CAP patients hospitalized in the same hospital with the use of multivariate regression, namely 83 patients in the observation study without ProADM (OPTIMA I) and the 169 patients in the intervention study (OPTIMA II RCT). **Results:** A total of 89 patients with confirmed CAP were included. As compared to patients with CURB65 only observed in the OPTIMA I study, adjusted regression analysis showed a significant shorter initial LOS (7.5 *vs.* 10.4 days; −2.32; 95% CI, −4.51 to −0.13; *p* = 0.04) when CURB65-A was used in clinical routine. No significant differences were found for LOS within 30 days. There were no significant differences in safety outcomes in regard to mortality and ICU admission between the cohorts. **Conclusion:** This post-study survey provides evidence that the use of ProADM in combination with CURB65 (CURB65-A) in “real life” situations reduces initial LOS compared to the CURB65 score alone without apparent negative effects on patient safety.

## 1. Introduction

Community-acquired pneumonia (CAP) is one of the most common infectious diseases associated with high morbidity, mortality and financial burden [1]. Different clinical risk stratification scores for management of CAP, prediction of mortality, and the need for hospitalization were developed in the last years and are recommended by guidelines [2]. Among limitations of clinical risk scores are their static behavior and poor memorability. In contrast biomarkers are objective, dynamic, and easily measurable. ProADM improved the prognostic accuracy of the pneumonia severity index (PSI) [3] and seemed to be a useful risk stratification tool [4]. In addition, the performance of the biomarkers ProADM and procalcitonin were comparable with the established clinical scores PSI and CURB65 [5,6,7]. We previously combined ProADM cut-offs with CURB65 classes to the CURB65-A score [8] and developed an algorithm to reduce and shorten hospitalizations in patients with low medical risk (OPTIMA I observation study) [9,10].

Hospitalization rates and length of stay (LOS) are affected by medical, biopsychosocial, and functional factors, as well as by the preferences of patients and their relatives [10,11,12,13]. Therefore, our algorithm included the post-acute care discharge score (PACD on admission and day three) [14] and the self-care index (SPI = “Selbstpflegeindex”, outpatients and during the ward stay) [15].

Nurse-led units (NLU) are already implemented in the United Kingdom and Scandinavia as institutional settings for patients with low medical but predominantly nursing care needs [16]. In nurse-led care nurses are responsible for the coordination and steering of patient care [9,10,17].

From 2010 to 2011, we conducted a single-center proof-of-concept randomized controlled trial (OPTIMA II RCT). 313 patients with LRTI were enrolled. This trial showed a trend for reduction of LOS during the initial encounter (0.5 days) and for overall hospitalizations (0.7 days) within 90 days in the ProADM-enhanced intervention group compared to the control group [17].

However, results from an RCT may not unconditionally be generalized because of exclusion criteria or non-enrollment and are frequently not adequately implemented in daily practice. Therefore, we performed a post-study surveillance to investigate the real-life effectiveness of our ProADM-enhanced algorithm for site of care decision in patients with CAP after completion of the OPTIMA II RCT. We also compared LOS with historic patient cohorts from the previous OPTIMA I and OPTIMA II RCT studies.

## 2. Methods

**Figure 1:**
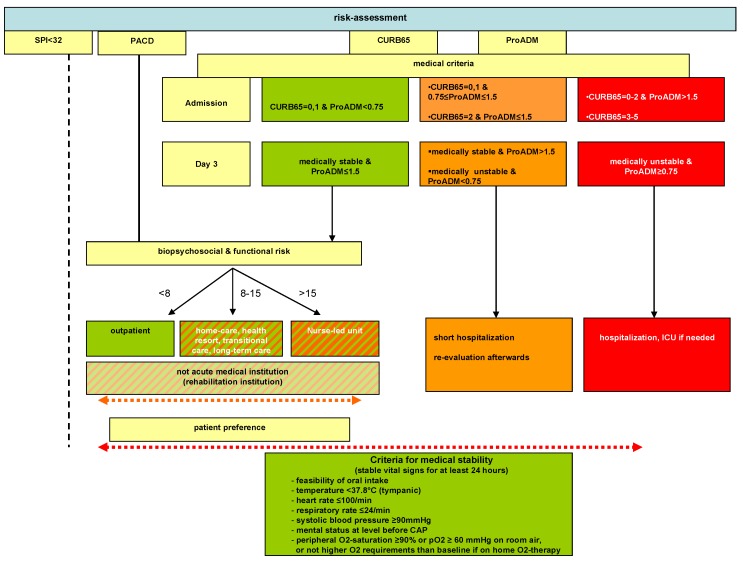
Algorithm of risk assessment for triage decisions on admission and during hospitalization.

Herein, we performed observational post-study quality surveillance at the Medical University Department of the public, cantonal Hospital of Aarau, a tertiary care 600-bed hospital in Northern Switzerland. Consecutive adult patients with CAP presenting to the ED were enrolled from September 2011 to April 2012. There were no exclusion criteria. Patients were registered on a password-secured website by the treating physician. All patients were triaged according to the algorithm consisting of medical (CURB65-A including ProADM on admission as described previously [8]), biopsychosocial (PACD), and functional (SPI) criteria (Figure 1).

Predefined medical, biopsychosocial, and organizational criteria and patient’s preference could be used to optionally overrule triage decisions and transfer patients to higher risk classes. Patient’s preference had priority for the final triage decision [17]. Treating physicians and nurses were reminded of correct application of the triage algorithm including ProADM values, stability, and overruling criteria. Nursing staff received ongoing training on correct use of biopsychosocial and functional criteria.

Medical stability was evaluated twice daily throughout hospitalization. The discharge management was also evaluated daily based on PACD, SPI, and the clinical judgment about need of social worker involvement. On day 3, additional ProADM values were used to reassign each patient to the appropriate risk class and PACD for the biopsychosocial risk according to the triage-algorithm. Patients were considered appropriate for discharge if stability criteria were fulfilled for 24 h during hospitalization (Figure 1).

PACD: Post-acute care discharge score; SPI: “Selbstpflegeindex” self-care deficit score; ProADM: Proadrenomedullin; ICU: Intensive care unit.

### 2.1. Medical Overruling Criteria

1. Admission to ICU, based on respiratory (respiratory rate ≥ 30/min and/or SO2 < 90% with 6 L O^2^/min) or hemodynamic instability (systolic blood pressure for ≥1 h <90 mmHg despite adequate volume resuscitation or vasopressor requirement);
2. Life-threatening co-morbidity, *i.e.*, imminent death; complications (abscess, empyema); for COPD GOLD III & IV; O^2^-saturation < 90% despite 30 min intensive treatment;
3. Acute illness requiring hospitalization independent from CAP;
4. Comorbidity, *i.e.*, immunodeficiency (neutrophils < 500/μL; if HIV+: CD4 < 350/μL, leukemia, lymphoma, myeloma, cytotoxic medications, hemodialysis), pneumonia within last 6 weeks, hospitalization independent of indication within the last week, other significant lung disease (cancer, fibrosis, bronchiectasis, tuberculosis, pulmonary embolism, cavitary lung disease);
5. Confusion, delirium, or intravenous drug use.

### 2.2. Biopsychosocial and Functional Overruling Criteria

1. Criteria requiring intensive nursing care, *i.e.*, dementia, recurrent falls, pressure ulcer, and inability to reliably take medications;
2. SPI score < 32 points in patients with a low PACD score (<8);
3. Deficit of mobility or self-care requiring treatment.

### 2.3. Organizational Overruling Criteria

1. Waiting for placement in a non-acute medical care facility (holiday bed, rehabilitation, nursing home, home health care);
2. Waiting for laboratory results, imaging studies or consultant examinations.

### 2.4. Patient’s Preference Overruling Criteria

1. Patient’s or relative’s concerns about safety at home;
2. Lack of supporting social network;
3. Financial reasons.

Site of care was determined by biopsychosocial and organizational factors in patients who were otherwise appropriate for discharge.

All patients underwent a standardized quality control phone interview on days 30 and 180. Informed consent was waived by the local ethics committee (EKAG 2010/045, EKAG 2009/074, Kantonale Ethikkommission Aargau).

ProADM was measured on admission, and on day 3, in the laboratory from EDTA plasma with a commercially available immunoassay (MR-ProADM, Thermofisher Scientific-BRAHMS AG, Hennigsdorf, Germany) with a functional assay sensitivity of 0.12 μg/L [4]. Results were routinely available around the clock within 1.5 h, upon ordering.

### 2.5. Definitions

CAP was defined as a new infiltrate on chest radiograph and at least one respiratory symptom and an auscultatory sign or a sign of systemic infection in patients with symptoms of a lower respiratory tract infection [18].

Patients were considered medically stable as described in Figure 1 [17].

### 2.6. End Points

The primary endpoint was LOS of index hospitalization and within 30 days. The secondary endpoints were intensive care unit (ICU) admission and all-cause mortality.

### 2.7. Statistics

Discrete variables were expressed as counts (percentage) and continuous variables as medians or means and standard deviations or interquartile range, unless stated otherwise. We used a linear regression model adjusted for sex, age, initial levels of procalcitonin and albumin, as well as the CURB65 score, to compare the LOS of index hospitalization and within 30 days of this cohort with the previously published cohorts from a prospective observational quality control study (OPTIMA I) and a randomized controlled trial (OPTIMA II RCT).

For the adverse outcomes death and intensive care unit (ICU) admission, logistic regression analyses were performed to assess the safety of the use of the algorithm.

Statistical analyses were performed using STATA version 12.1 (Stata Corp, College Station, TX, USA). All the testing was 2-tailed, and *p* < 0.05 was considered statistically significant.

## 3. Results

### 3.1. Baseline Characteristics

A total of 115 patients with CAP were enrolled in this study, 25 had a final diagnosis other than CAP, and one patient was included twice. Therefore, we analyzed a total of 89 patients with CAP.

We compared our results with the 83 patients of the OPTIMA I observation study and the 169 patients of the OPTIMA II intervention study with CAP as final diagnosis who completed 30 days follow-up; four patients had been lost to follow-up between day 30 and day 90 and had therefore not been included in the previously published article [17]. Baseline characteristics in these groups are shown in Table 1.

**Table 1:** Baseline characteristics.

Demographic Characteristics	Observation with ProADM	Intervention		Observation without ProADM
	(*n* = 89)	without ProADM (*n* = 93)	with ProADM (*n* = 76)	(*n* = 83)
Mean age (years, range)	64.3 (18–94)	63.9 (18–93)	67.6 (22–92)	64.0 (16–93)
Sex (male), No. (%)	52 (58.4%)	56 (60.2%)	49 (64.5%)	47 (56.6%)
*Initial treatment site, No. (%)*				
Inpatient treatment	85 (95.5%)	81 (87.1%)	55 (72.4%)	76 (91.6%)
Outpatient treatment	4 (4.5%)	12 (12.9%)	21 (27.6%)	7 (8.4%)
*Risk assessment*				
CURB65 class (mean; median)	1.6/1	1.7/1	1.7/1	1.9/2
CURB65 I	54	51	40	35
CURB65 II	20	24	19	17
CURB65 III	15	18	17	31
CURB65-A class (mean; median)	2.2/2	not applicable	2.2/2	2.2/2
CURB65-A I (No.)	13	not applicable	12	16
CURB65-A II (No.)	48	not applicable	35	32
CURB65-A III (No.)	28	not applicable	29	35
Charlson comorbidity index (mean)	4.6	3.9	3.8	not available
Heart rate (bpm)	98	96	94	100
Temperature (°C)	38	38	38	38.1
Systolic blood pressure (mmHg)	129.5	127.9	128.4	125.0
Respiratory rate (/min)	21.0	20.6	20.7	22.0
*Laboratory findings (mean)*				
Proadrenomedullin (nmol/L) (admission) (mean, range)	1.8 (0.4–12.2)	1.6 (0.5–10.4)	1.9 (0.4–22.1)	1.3 (0.4–15.1)
Proadrenomedullin (nmol/L) (d3) (mean, range)	2 (0.3–24.6)	1.2 (0.4–4.6) (14 missing)	1.4 (0.1–5.0) (13 missing)	not done
Procalcitonin (μg/L) (admission), (mean, range)	3.1 (0.06–101)	5.5 (0.06–170)	3.4 (0.06–79.2)	4.4 (0.06–58.4)
PCT < 0.25 (in %)	38.2 (34/89)	43.0 (40/93)	44.7 (34/76)	25.3 (21/83)
PCT 0.25–0.5 (in %)	21.4 (19/89)	20.4 (19/93)	17.1 (13/76)	18.1 (15/83)
PCT > 0.5 (in %)	40.4 (36/89)	35.6 (34/93)	38.2 (29/76)	56.6 (47/83)
C-Reactive protein, mg/L	143.9	144.9	148.6	not available
Leukocyte count, cells/μL	12.8	13.4	12.8	not available

### 3.2. Allocation to Treatment Site according to Triage Algorithm

Thirteen of 89 patients (14.6%) of the post-study surveillance OPTIMA III had a low medical risk according to the CURB65-A score (CURB65-A class I) and qualified for outpatient treatment at home with or without home health care, treatment in a non-medical care center (health resort, rehabilitation, nursing home) or in the NLU. Forty-eight of 89 patients (53.9%) were assigned to the intermediate risk group (CURB65-A class II) qualifying for a short-term hospitalization. Twenty-eight of 89 patients (31.5%) were assigned to the high-risk group (CURB65-A class III) who were supposed to be hospitalized.

Of the 13 patients in the low medical risk group, only three were treated as outpatients due to the presence of biopsychosocial and organizational criteria or patient’s preference. One patient in the intermediate medical risk group was treated as outpatient as well.

### 3.3. Historic Comparison

#### 3.3.1. Length of Stay

**Figure 2:**
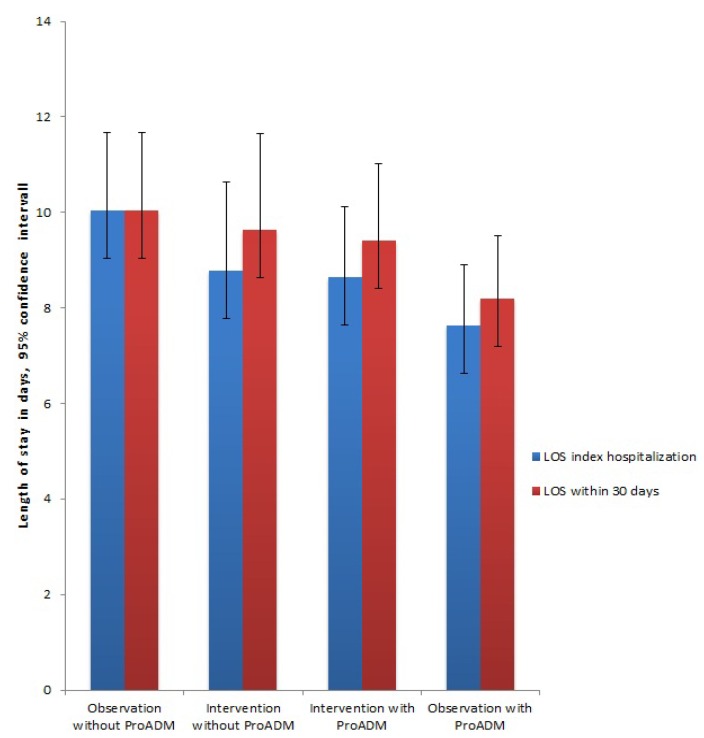
Length of stay during index hospitalization and within 30 days after admission.

We compared the length of stay within 30 days after enrollment in the patients of OPTIMA III observation study with two well-defined historic cohorts of CAP patients hospitalized in the same hospital from OPTIMA II RCT and OPTIMA I studies. Adjusted regression analysis for age, sex, initial levels of procalcitonin and albumin, as well as the CURB65 score, showed a significantly shorter LOS during index hospitalization in patients of OPTIMA III study compared to OPTIMA I (7.5 *vs.* 10.4 days; adjusted regression coefficient, −2.32; 95% CI, −4.51 to −0.13; *p* = 0.04). Regarding the initial LOS no significant differences were found for comparisons with OPTIMA II RCT intervention group (7.5 *vs.* 8.4 days; adjusted regression coefficient, 0.07; 95% CI, −2.16 to 2.3; *p* = 0.95) and control group (7.5 *vs.* 8.7 days; adjusted regression coefficient, −0.94; 95% CI, −3.06 to 1.17; *p* = 0.38). With the use of the CURB65-A score in our triage algorithm there was a non-significant trend for shorter LOS within 30 days compared to the OPTIMA I observation without ProADM (Table 2, Figure 2).

**Table 2:** Efficacy and safety outcome in the observation with ProADM (OPTIMA III) compared to former studies. All analyses are adjusted for age, sex, CURB65, albumin, and procalcitonin.

LOS during index hospitalization		
**Study**	**Regression Coefficient (95% CI)**	***p* Value**
Intervention without ProADM	−0.94 (−3.06 to 1.17)	0.379
Intervention with ProADM	0.07 (−2.16 to 2.3)	0.952
Observation without ProADM	−2.32 (−4.51 to −0.13)	0.038
**LOS within 30 days**		
**Study**	**Regression Coefficient (95% CI)**	***p* Value**
Intervention without ProADM	−1.2 (−3.41 to 1.02)	0.288
Intervention with ProADM	−0.11 (−2.45 to 2.23)	0.926
Observation without ProADM	−1.84 (−4.14 to 0.46)	0.116
**Mortality**		
**Study**	**Adjusted OR (95% CI)**	***p* Value**
Intervention without ProADM	0.47 (0.13 to 1.68)	0.247
Intervention with ProADM	0.65 (0.2 to 2.09)	0.465
Observation without ProADM	0.35 (0.09 to 1.29)	0.115
**ICU admission**		
**Study**	**Adjusted OR (95% CI)**	***p* Value**
Intervention without ProADM	0.48 (0.15 to 1.53)	0.214
Intervention with ProADM	0.43 (0.12 to 1.52)	0.189
Observation without ProADM	0.65 (0.22 to 1.92)	0.440

#### 3.3.2. Mortality and ICU Admission

With the use of the ProADM-enhanced algorithm, there was no significant increase of mortality within 30 days, as shown in the logistic regression model in Table 2.

The rate of patients admitted to the ICU was the same in the three studies, as shown in Table 2.

## 4. Discussion

The aim of our study was to describe the effects of biomarker-enhanced triage decisions over time on clinical outcome and length of stay during a randomized controlled trial and after the effort of its implementation into clinical routine. As previously described, we developed the algorithm with the observational cohort of OPTIMA I study [9] and introduced the algorithm in our center in the study setting of the OPTIMA II study [17]. In the historic comparison, we analyzed only patients with CAP to avoid the heterogeneity of patients with non-pneumonic lower respiratory tract infections (LRTI). There was likely a learning process, and over time, confidence in applying the algorithm in clinical care was established, after the treating physicians and nurses had gained personal experience and were provided with the results of the randomized controlled trial. This might not only relate to the use of ProADM alone but also to greater attention to the discharge process itself with confidence from early and safe discharges. Aujesky *et al.* showed that the adherence to the recommendations according to the PSI risk score for site of treatment decisions was low [19]. Similarly, Karmakar *et al.* [20] showed only 5% application of the CURB65 score in a New Zealand hospital. Our group showed already in the international multicenter ProREAL post-study surveillance that after testing of an algorithm in the ProHOSP RCT, the clinical routine could be changed in the participating centers [21]. Hansson *et al.* [22] showed in case of acute appendicitis that the results of a clinical trial can lead to a change in clinical practice. After initially overruling an antibiotic-only-algorithm by performing primary appendectomy in almost 50% of the time during a randomized trial [22], surgeons in the same hospital network changed their practice to primarily only use antibiotics in 79% of patients after this was shown to be successful in the previous randomized trials [23].

However, in contrast to the reduction in overall LOS, there remained reluctance by the treating physicians to discharge patients from the emergency room and to treat patients entirely as outpatients.

Our main finding in the historic comparison was a significantly shorter LOS in the observational cohort with ProADM guidance (OPTIMA III) compared to the observational cohort without ProADM guidance (OPTIMA I). The comparison between the current observational study, using ProADM guidance, with the OPTIMA II RCT study results is more complicated as several aspects need to be considered. Overall, there was no difference compared to either the OPTIMA II intervention group or the OPTIMA II control group. As previously stated in the discussion part of the OPTIMA II article [17], the OPTIMA II RCT used an interdisciplinary risk assessment bundle and compared it with a highly competitive, guideline-conforming and strictly reinforced control group, which by itself optimized LOS. In contrast, the current OPTIMA III study was an observational cohort without exclusion criteria and without reinforced algorithm adherence and even though we controlled for age, sex, initial levels of procalcitonin and albumin, as well as the CURB65 score, we cannot exclude unmeasured differences between the two populations or differences due to other unmeasured confounders. On the other hand, there was likely a learning effect with greater experience and confidence from the results of the previously RCT. Therefore, and in view of the lack of a significant difference even between the intervention group and the control group within the OPTIMA II RCT, it is not surprising to find no difference between LOS in OPTIMA III and the OPTIMA II control group.

Moreover the non-significant trend for increased mortality is unlikely related to the implementation of our algorithm but rather a selection bias of differential patient populations included in a clinical trial and in a post-study surveillance. In OPTIMA III, we included patients with severe comorbidities and immunodeficiency including terminally ill patients who were formerly excluded in the RCT. With adjustment for known confounders for mortality, there was no significant increase of mortality over time.

There were no major structural, organizational or strategic alterations in our hospital, but from 2009 to 2012 there was a trend for shorter LOS 30 days after enrollment and even significantly shorter LOS of initial hospitalization with the use of the CURB65-A score. Of note, diagnosis related groups (DRGs) have been introduced in Switzerland in January, 2012, but a similar financing system had already been in place for years in our canton throughout the observed time periods.

We show the development, implementation and use of the algorithm with proadrenomedullin enhanced CURB65 score over four years in our publications. The description of the derivation of the biomarker enhanced score, clinical trial, and post-study surveillance could be used as a model for new clinical scores and algorithms in LRTI and other diagnosis [24,25]. The studies (OPTIMA I–III) had well characterized cohorts, similar methodology and overall 341 patients with radiologically confirmed CAP.

## 5. Potential Limitations

The study was performed in a single centre, and the results were compared to previous studies from the same centre. Therefore, our results have a lack of generalizability. Another limitation is the small sample size. We focused on patients with radiologically confirmed pneumonia in order to avoid heterogeneity. Prior to widespread implementation, our algorithm has to be tested in different settings, ideally in multicenter studies with sufficient sample sizes.

## 6. Conclusions

In our study, we show that the implementation of a biomarker-enhanced triage algorithm in clinical routine was feasible. Effective and early triage for site of care and a timely discharge after hospital admission are important to avoid nosocomial complications and reduce healthcare costs in the time of DRGs. Our studies contribute to the development of biomarker-guided and safe triage algorithms with objective criteria. These promising results are the basis for further research to improve triage decisions [26].
